# Cost-Benefit Analysis of an Enhanced Recovery Program for Gastrectomy A Retrospective Controlled Analysis

**DOI:** 10.1007/s00268-021-06220-2

**Published:** 2021-08-07

**Authors:** Valentine Luzuy-Guarnero, Caroline Gronnier, Sergio Figuereido, Styliani Mantziari, Markus Schäfer, Nicolas Demartines, Pierre Allemann

**Affiliations:** 1 Department of Visceral Surgery, Centre Hospitalier Universitaire Vaudois (CHUV), Rue du Bugnon 46, CH-1011 Lausanne, Switzerland; 2 Digestive Surgery, CHU of Bordeaux, Bordeaux, France

## Abstract

**Background:**

Enhanced recovery programs (ERP) demonstrated decreased postoperative complication rate and reduced length of stay (LOS). Recently, data on the financial impact revealed cost reduction for colorectal, liver and pancreatic surgery. The present study aimed to assess the cost-effectiveness of ERP in gastric surgery.

**Methods:**

ERP based on enhanced recovery after surgery *(*ERAS®) society guidelines was implemented in our institution, in June 2014. Consecutive patients undergoing gastric surgery after ERP implementation (*n* = 71) were compared to a control group of consecutive patients operated before ERP implementation (*n* = 58). Primary endpoint was cost-effectiveness including detailed perioperative costs. Secondary endpoints were postoperative complications and LOS. Standard statistical testing (means, Mann–Whitney Fisher’s exact T test or Pearson Chi-square test) was used.

**Results:**

Both groups were comparable regarding demographic details. Mean (SD) overall costs per patient were lower in the ERP group (€33,418 (17,901) vs €39,804 (27,288), *P* = 0.027). Lower costs were found for anesthesia and operating room (−€2 356), intensive or intermediate care (−€8 629), medication (−€1 196)), physiotherapy (−€611), laboratory (−€1 625)) and blood transfusion (−€977). Overall complication rates in ERP and control group (51% vs 62%, *P* = 0.176) were similar. Mean length of stay (SD) (14(13) days vs 17(11) days, *P* = 0.037) was shorter in the ERP group.

**Conclusion:**

ERP significantly reduces overall, preoperative and postoperative costs in patients undergoing major gastric surgery.

## Introduction

Since the 1990s, various and not always well-defined fast tracks or enhanced recovery protocols (ERP) were promoted worldwide. They consist in multimodal perioperative management programs, aiming to improve postoperative recovery [1]. Evidence-based guidelines were implemented for colon and rectal surgery and for both pancreatic and liver surgery [2–8]. For these types of surgery, many studies demonstrated substantial benefits like reduced length of stay (LOS) and reduced complications rate [9–14].

Recently, the ERAS® Society published guidelines for gastrectomy [15]. Due to limited experience, data remain scarce. Two recent meta-analyses, including only preliminary data, showed promising results, mainly shorter LOS without increased complications [16, 17].

In addition, minimally invasive surgery (MIS) for gastrectomy is now validated and established and some studies suggested that combining ERP protocol with MIS could improve postoperative recovery and early postoperative nutritional status as well as reduce postoperative stress reaction [18–20].

Currently, reducing costs is increasingly a major issue for national healthcare systems. ERPs have demonstrated to reduce the costs for colorectal [21], pancreatic [22] and liver surgery [23]. The question remained open for gastric surgery. Kim et al. [24] showed no differences in hospital charges. However, costs were not the primary endpoint of the study and the number of patients was low. More recently, Liu et al. [20] showed significantly lower charges, but only medical costs were assessed.

The aim of the present study was to analyze the complete cost-effectiveness of ERP in gastric surgery. Secondary endpoints were to assess LOS and morbidity rate.

## Methods

An ERP for gastric surgery based on the recommendations of the ERAS® Society [15] was implemented in our department in June 2014. The detail of the various pre-, intra- and postoperative elements included in the protocol is listed in Table 1. The medical and nursing staff were already familiar with ERAS® protocols and pathways, since such protocols were previously implemented for colorectal, pancreatic and liver surgery in 2011, 2012 and 2013, respectively. Furthermore, the dedicated ERAS® team which included surgeons, anesthetists, nurses and nutritionists did already exist and was well trained. The study protocol was approved by the local Ethical Committee (CER-VD, protocol number: 2016-01,075). Moreover, all patients had signed the institutional general informed consent for clinical research.

**Table 1:** Perioperative care used before and after the introduction of the ERP

	ERP	Control group
Patient counseling and education	Preadmission counseling and written information	No standardized information
Fasting	Clear fluids until 2 h, solids 6 h before surgery	Solid and fluid fasting from midnight
Carbohydrate drinks	800 ml on evening and 400 ml 2 h before surgery	None
Premedication	No premedication	At discretion of the anethetist
Thromboprophylaxis	LMW heparin 12 h before surgery, IPC	LMW heparin 12 h before surgery
PONV prophylaxis	Droperidol + ondansetron ± betamethasone	Not routinely used
Hypothermia prevention	Active warming with air blanket	Active warming with air blanket
Antibiotic prophylaxis	Cefuroxime 1.5 g at induction	Cefuroxime 1.5 g at induction
Balanced intraveinous fluids	Near-zero fluid balance. Balanced crystalloids should be preferred to 0,9% saline. Postoperative crystalloïds 1000 ml during the first 24 h, then stop	No guidance
Postoperative analgesia	Epidural for laparotomy. Paracetamol, metamizole and oxycodone-naloxone (when epidural is removed)	No standardized postoperative analgesia
Abdominal drains	No routine abdominal drainage	Abdominal drainage at surgeon's discretion
Nasogastric tube	Total resection: Feeding NGT removed on POD 5	Systematic NGT, removed at surgeon's discretion
	Partial resection: No NGT	
Urinary catheter	Removed on POD 2	Removed at surgeon's discretion
Postoperative nutrition	Fluid 4 h after surgery (max 1L/24 h), enteral nutrition 250 ml/24 h through NGT from day of surgery, free fluids (whithout gaz) and 2 nutritional supplements per day from POD 1	No standardized reintroduction of fluids and food
	Subtotal resection: light meal on POD 2, normal meal on POD 3	
	Total resection: mixed light meal and enteral nutrition 500 ml/24 h on POD 3, light meal on POD 4	
Glycemic control	Insulin protocol in the event of hyperglycemia	Not routinely used
Laxatives	Not routinely used	Not routinely used
mobilization	Out of bed for at least 2 h on day of surgery; at least 8 h from day 1	No protocol
Systematic audit	Audit meeting every 2 months	None

*LMW* low molecular weight; *IPC* intermittent pneumatic compression; *PONV* postoperative nausea and vomiting; *NGT* nasogastric tube; *POD* postoperative day

### Patient selection

A prospective ERP cohort of consecutive patients was compared with a retrospective control group (standard care). All consecutive patients with total or partial gastrectomy from June 2014 to December 2019 were included in the ERP group. The control group included all consecutive patients operated from January 2010 to May 2014 before ERP implementation. There was no selection based on the pathological condition (benign or malignant). However, patients with any kind of wedge gastric resection or gastrectomy as part of a multivisceral resection and patients with missing cost-related data were excluded.

According to the study of Staiger et al. [25], patients with a high overall postoperative morbidity index are prone to induce significant selection bias, particularly in costs analysis. For this reason, we decided to exclude patients with several Grade IIIb or IV complications leading to a Comprehensive Complication Index (CCI) [26] higher than 50 from the cost’s analysis.

### Assessment of postoperative outcomes and discharge criteria

Postoperative complications were graded according to the Clavien classification [27]. Grade IIIa–IVb were defined as major complications. Postoperative mortality (grade V) was defined as death during the first 30 days after the index operation or during the hospital stay [27]. The CCI was also calculated for each patient.

The LOS was calculated from the day of the operation to the day of discharge from hospital. Finally, compliance rate was calculated in the ERP group as the average of the compliance to each ERAS® items (Table 1), which correspond to the number of fulfilled ERAS® items divided by the total number of items.

### Cost analysis

Detailed costs for each patient were extracted from the hospital accounting database via the administration service. Costs were calculated until the day the patient leaves the hospital. They were divided into intraoperative and preoperative/postoperative costs. Intraoperative costs included costs from anesthesia, operation room (OR) and disposable materials used in the OR. Anesthesia costs were calculated per minute based on the duration of anesthesia (including costs of anesthetists, nurse anesthetists, materials and drugs). OR costs were based on the OR occupation in minutes at our institutional standard rate. Preoperative and postoperative costs included: intensive care unit (ICU) and intermediate care unit (IC) costs, calculated per day, based on the Project Research in Nursing (PRN) score [28], medical care, nursing care, physiotherapy, medication, blood transfusion and testing, laboratory test, radiology, pathology, housing, administration and other (social work, priest and occupational therapy). Medical care included all clinical activities performed by doctors, including surgeon’s costs and the costs of other non-operative procedures (endoscopy, drainage as example). Nursing care costs were measured per day outside intensive and intermediate care units, based on the PRN score. Costs of housing were counted per day, whereas administration costs were counted per patient admission.

### Cost-minimization analysis

The cost-minimization analysis was performed from a healthcare provider’s perspective to assess savings per patient in hospital. This analysis corresponded to the subtraction of the control group costs per patient to the ERP costs per patient and the ERP-specific costs per patient. The ERP-specific costs included the patient’s logbook (€5), the preoperative carbohydrate drinks and finally the nutritional supplements (€14). These costs (total of €19) were low and thus considered as negligible. Costs were obtained in Swiss francs (CHF) and then converted to euros (€). The exchange rate used was 1CHF = 0,93€, which was the official rate on June 10, 2020.

### Statistical analysis

Continuous variables were compared using Mann–Whitney U test, whereas discrete variables were analyzed by means of Fisher’s exact T test or Pearson Chi-square test. The arithmetic mean was considered as the most informative measure from a pharmaco-economic point of view. A *p* value < 0.05 was considered statistically significant. All analyses were performed using STATA® software (ver 16).

## Results

### Patient characteristics

**Fig. 1:**
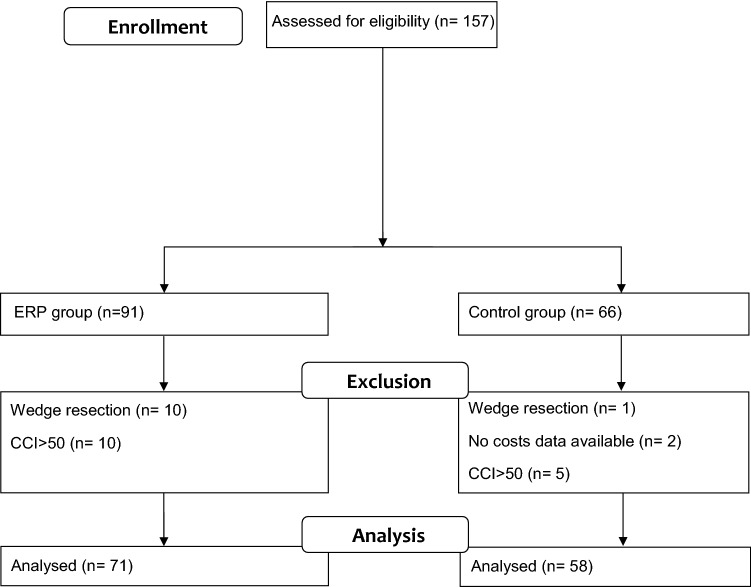
CONSORT flow chart

During the study time, 157 gastric procedures were performed. Eleven wedge resections were excluded according to our inclusion/exclusion criteria. Hence, 144 patients met the inclusion criteria (see CONSORT flowchart, Fig. 1). Two patients had to be excluded in the control group, due to the lack of cost-related data. In the final analysis, ERP group included 81 patients and control group 63 patients. Both groups were similar in terms of demographics, co-morbidities and surgical characteristics except for the surgical approach (laparoscopy was more frequently performed in the ERP group (*P* < 0.001). (Table 2).

**Table 2:** Patient demographics and surgical details

	ERP group (*n* = 71)	Control group (*n* = 58)	*P* value
Age (years)*	60.6 (14)	61.5 (14)	0.784^†^
Sex ratio (M:F)	42:29	37:21	0.591^§^
*ASA grade*			
I-II	50	58	
III-IV	21	0	
Charlson Index	0.6 (1)	0.6 (1)	0.895^†^
*Surgical approach*			
Laparoscopic	41	4	** < 0.001**^§^
Open	25	53	** < 0.001**^§^
Converted	5	1	0.154^§^
*Type of resection*			
Total gastrectomy	42	36	0.591^§^
Partial gastrectomy	26	21	0.961^§^
Other	3	1	0.415^§^
*Diagnosis*			
Malignant disease	66	51	0.328^§^
Benign disease	5	7	
AJCC Stage			0.758^§^
I	16	10	
II	13	10	
III	20	21	
IV	9	6	
Preoperative chemotherapy	40	25	0.135

Values are *mean(s.d.). † Mann–Whitney test. § Pearson Chi-square test

Bold value indicates statistical significance (*p* < 0.05)

*AJCC* American Joint Committee on Cancer

Five patients presented a CCI > 50 in the control group and 10 in the ERP group. These 15 patients represented 10% of the total sample size and were excluded from the costs analysis as stated in the method section.

### Costs analysis

Costs details are shown in Table 3. The mean total costs were €39 804 for the control group and €33 418 for the ERP group (*P* = 0.027). The mean intraoperative costs per patient were €11 613 for the control group and €11 141 for the ERP group (*P* = 0.839). The mean pre- and postoperative costs were €28 135 and €22 276 for control group and ERP group, respectively (*P* = 0.014). ERP was associated with lower costs for almost all items except for the disposable materials and the nutritionists. The difference of the mean total costs per patient between the two groups was €6 386 (-36%) in favor of the ERP group.

**Table 3:** Detailed costs for the ERP and the standard group in euros

	Mean cost per patient (€)			
	ERP group* (*n* = 71)	Control group* (*n* = 58)	Difference (ERP-Control group)	*P* value†
Total intraoperative costs	11,141 (3195)	11,613 (28,191)	− 472	0.839
Disposable materials	4029 (1769)	2917 (1901)	1111	** < 0.001**
Anesthesia and operating room	7113 (1992)	8696 (3787)	− 1584	**0.001**
Total preoperative + postoperative costs	22,276 (16,572)	28,135 (24,527)	− 5859	**0.014**
ICU/IC	3078 (4832)	6151 (8615)	− 3073	** < 0.001**
Medical care	4931 (2757)	5207 (3232)	− 276	0.489
Nursing care	7049 (6786)	7093 (6043)	− 44	0.340
Medication	357 (834)	723 (695)	− 365	** < 0.001**
Physiotherapy	300 (411)	546 (796)	− 246	** < 0.001**
Radiology	535 (689)	837 (1250)	− 302	**0.024**
Laboratory	764 (771)	1531 (1900)	− 766	** < 0.001**
Pathology	2305 (997)	2442 (1155)	− 137	0.751
Blood transfusion and testing	127 (292)	882 (2485)	− 754	** < 0.001**
Housing	2181 (1800)	2147 (1852)	33	0.501
Interventional radiology and endoscopy	305 (644)	310 (620)	− 5	0.605
Perioperative disposable material	265 (767)	339 (966)	− 73	0.389
Nutritionists	189 (310)	52 (149)	137	** < 0.001**
**Total costs**	**33,418 (17,901)**	**39,804 (27,288)**	− **6386**	**0.027**

Values are *mean(s.d.). † Mann–Whitney test. Bold value indicates statistical significance (*p* < 0.05)

*ICU* Intensive care unit, *IC* intermediate care unit. *CCI* Comprehensive complication index

### Perioperative outcomes

Perioperative data are summarized in Table 4. Duration of operation as well as duration of anesthesia was significantly shorter in the ERP group (*P* < 0.001 and *P* = 0.001, respectively). Moreover, there was less blood loss in the ERP group (*P* < 0.001) and the length of stay was shorter in the ERP group (*P* = 0.026). On the other hand, the two groups were similar in terms of overall complication rate (*P* = 0.176), minor complication rate (27% vs 26%, *P* = 0.977), major complication rate (35% vs 23%, *P* = 0.130), median CCI (22.1 vs 19.6, *P* = 0.320) and reoperation rate (16% vs 10%, *p* = 0.977). The overall rate of compliance to the ERAS protocol was 88%.

**Table 4:** Perioperative outcomes

	ERP group* (*n* = 71)	Control group* (*n* = 58)	*P* value
Duration of operation (min)	207 (63)	249 (55)	** < 0.001**^†^
Duration of anesthesia (min)	280 (79)	322 (62)	** < 0.001**^†^
Blood loss (ml)	173 (224)	392 (347)	** < 0.001**^†^
Complications			
Minor (I-II)	21	24	0.162^§^
Major (III-IV)	11	34	** < 0.001**^**§**^
Death (V)	0	0	0.376^§^
CCI	12.9 (15.6)	17.9 (17.2)	0.088^†^
Reoperation	3	10	**0.015**^§^
LOS	14 (13)	17 (11)	**0.026**^†^
Readmission	10	4	0.173

Values are *mean(s.d.). † Mann–Whitney test. § Pearson Chi-square test

Bold value indicates statistical significance (*p* < 0.05)

*CCI* Comprehensive complication index, *LOS* Length of stay

### Subgroup analysis

A subgroup analysis was performed by weighing the costs according to the CCI. Results are shown in Table 5.

**Table 5:** Costs according to CCI

	Mean cost per patient (€)			
	ERP group*	Control group*	Difference (ERP – Control group)	*P* value†
CCI 20	25,940 (12,176)	29,253 (6199)	− 3313	**0.014**
CCI 30	28,045 (12,493)	30,961 (8070)	− 2916	**0.017**
CCI 40	31,899 (17,568)	32,561 (9341)	− 662	**0.048**
CCI 50	33,418 (17,901)	39,804 (27,288)	− 6386	**0.027**

Values are *mean(s.d.). † Mann–Whitney test

Bold value indicates statistical significance (*p* < 0.05)

*CCI* Comprehensive complication index

## Discussion

The present study shows that the implementation of an ERP protocol for gastric surgery allows a significant reduction of costs of €6 386 per patient, in patients with moderate number of complications (CCI ≤ 50). Moreover, higher costs reduction was observed as the complication rates decrease, as shown in Table 5. Those results corroborate those of the study of Staiger et al. [25]. Their prediction model showed that each 10-point increase in CCI corresponded to a 14% increase to the baseline cost.

Our observations confirm the recent meta-analysis by Chen et al. [29] that included seven randomized controlled trials (RCTs), where hospitalization costs were found to be reduced in ERP group. In the present study, analyses of costs were more detailed and displayed significant savings in various type of care. Total mean costs were separated in two main categories: total intraoperative costs and total preoperative + postoperative costs. This second category of costs was significantly lower in the ERP group and included the majority of savings. The first one was the ICU/IC costs. The majority of patients included in the ERP group did not stay in the ICU and spent just a few days in the IC compared to the control group, which is an interesting outcome of ERP per se. One of the reasons is probably the introduction of standardized anesthesia protocol and standardized care maps, guiding the postoperative period, thus decreasing monitoring needs and increasing patient’s autonomy. The second main gain was in medication and laboratory tests. This is also explained by the care-maps use, leading to the elimination of unnecessary laboratory tests and a standardized postoperative medication plan. A nonsignificant reduction in costs for medical and nursing care was observed in the ERP group. This could be attributed to similar complication rate, since that factor strongly drive expenditures. Conversely, the costs for disposable materials were significantly higher in the ERP group. This is most likely linked with the more frequent use of laparoscopy. Moreover, with the introduction of ERP protocols, increased attention was paid to nutritional status with increased nutritional monitoring and thus increased costs of nutritionists.

The shorter LOS in the ERP group (-3 days, *P* = 0.026) is consistent with data present in the literature [19, 20]. ERAS® pathway has shown to reduce LOS [30] in colorectal [10, 13], pancreatic [22] and liver surgery [23]. In addition, several studies showed similar results for gastric surgery [16, 17, 29, 31]. A recent meta-analysis including six RCTs showed a shorter LOS of 2.65 days for the ‘fast-track’ groups (*P* < 0.001) [16]. Of note, our LOS is globally longer than reported in the literature. Selection of patients is the major reason for that. In fact, when looking at the inclusion/exclusion criteria of these studies, we can observe that patients > 75 years, with an American Society of Anesthesiologists (ASA) score of III or IV as well as total gastrectomy for advanced stage cancer, are most often excluded. In our study, we included all patients, and age, pre-existing co-morbidities or advanced cancer stage were not considered as exclusion criteria.

On the other hand, we observed discordant results regarding complication rate. In the present study, the complication rate (both minor and major) was similar as in the meta-analysis of Li et al. [16]. However, ERAS® pathway has commonly shown to reduce complication rate in colorectal [10, 13], pancreatic [22] and liver surgery [23]. Our nonsignificant results concerning morbidity could be explained by the fact that ERP was already implemented in our department many years ago in several different surgeries (colorectal, pancreatic, and liver). As suggested by a previous study in liver surgery [32], this may have influenced both surgeons and care givers for the control group.

Finally, our results confirm those of the meta-analysis of Yu et al. [31], which showed a 2-day LOS decrease (*P* < 0.001) and a decrease in costs of $506 (*P* < 0.001) in ‘fast-track’ patients, with a comparable complication rate and readmission rate.

As shown in the literature, implementation of ERP protocols needs an initial consequent investment, but is later associated with important cost reductions [10, 22, 23, 33]. In our department, ERAS® was implemented in 2011 for colorectal surgery and gradually applied to other types of abdominal surgery. Costs of education and training of each team-member were therefore granted. Introducing new ERPs for other types of surgery in the same department will decrease more and more the implementation costs that may finally become negligible.

The present study has some limitations that need to be addressed. The most important limitation is the increased use over time of laparoscopy in gastric surgery, clear bias in favor of ERP patients, as showed in the literature [34]. Another limitation is the difficulty to differentiate between the pathophysiological effects of the ERP protocol and benefits of standardization itself. Moreover, the retrospective design with subsequent missing data could induce some bias. However, a prospective randomized trial seems today not ethical, as ERAS® pathway has become standard of care in our department, with proven benefits for patients and their outcome. Moreover, a randomization within a surgical department with ERAS protocols in other surgical specialties is not possible, as prior ERAS habits significantly influence outcome in the control group [32].

On the other hand, an important strength of the present study is its detailed real costs analysis. This real cost analysis may allow to better understand some specific aspects of perioperative care influenced by the implementation of an enhanced recovery program.

## Conclusion

ERP is cost-effective in gastric surgery patients, with higher costs savings in patients with no or limited postoperative complications.
